# Imperatives for DUCHENNE MD: a Simplified Guide to Comprehensive Care for Duchenne Muscular Dystrophy

**DOI:** 10.1371/currents.md.87770501e86f36f1c71e0a5882ed9ba1

**Published:** 2015-08-07

**Authors:** Kathi Kinnett, Sunil Rodger, Elizabeth Vroom, Pat Furlong, Annemieke Aartsma-Rus, Kate Bushby

**Affiliations:** Parent Project Muscular Dystrophy, Hackensack, New Jersey, USA; TREAT-NMD, Newcastle University, Newcastle upon Tyne, UK; United Parent Project Muscular Dystrophy (UPPMD), Veenendaal, the Netherlands; Parent Project Muscular Dystrophy, Hackensack, New Jersey, USA; United Parent Project Muscular Dystrophy (UPPMD), Veenendaal, the Netherlands; TREAT-NMD, Newcastle University, Newcastle upon Tyne, UK; TREAT-NMD, Newcastle University, Newcastle upon Tyne, UK

## Abstract

Duchenne muscular dystrophy (DMD) is a progressive, life-limiting muscle-wasting disease. Although no curative treatment is yet available, comprehensive multidisciplinary care has increased life expectancy significantly in recent decades. An international consensus care publication in 2010 outlined best-practice care, which includes corticosteroid treatment, respiratory, cardiac, orthopedic and rehabilitative interventions to address disease manifestations. While disease specialists are largely aware of these care standards, local physicians responsible for the day-to-day care of patients and families may be less familiar. To facilitate optimal care, a one-page document has been generated from published care recommendations, summarizing the key elements of comprehensive care for people living with DMD (“Imperatives for Duchenne muscular dystrophy). This document was developed through an international collaboration between Parent Project Muscular Dystrophy (PPMD), United Parent Projects Muscular Dystrophy (UPPMD) and TREAT-NMD.

## Introduction

The muscular dystrophies (MD) are a group of genetically heterogeneous muscle diseases marked by progressive wasting and weakness of the skeletal and cardiac muscles^1^. Duchenne muscular dystrophy (DMD) is the most common and most severe form. It is an X-linked disorder affecting 1 in 5000 live male births^2^^,^ 3. DMD is caused by mutations in the DMD gene, which encodes the muscle fiber membrane protein dystrophin. Deficiency or complete absence of dystrophin makes muscle fibers sensitive to damage upon contraction, leading to plasma membrane leakage and muscle fiber degeneration, which eventually leads to progressive muscle degeneration and loss of ambulation^4^.

The average age at diagnosis of Duchenne is approximately five years, but delays in motor milestones (such as sitting, standing independently, climbing, and walking), and cognitive development (such as expressive language, receptive language, visual-spatial skills, attention and memory) occur much earlier^5^^,^ 6. Untreated, children with DMD lose their ability to walk independently and become reliant on wheelchairs for mobility between the ages of 7 and 13. Most individuals with Duchenne experience serious respiratory, orthopedic, and cardiac complications. By the age of 18, the majority of patients require ventilation support at night^7^^,^ 8. The average life expectancy is approximately 30 years^7^, with respiratory complications and cardiomyopathy being common causes of death.

Standard management of Duchenne requires multidisciplinary care that includes the use of corticosteroids as well as respiratory, cardiac, orthopedic, and rehabilitative interventions addressing both the primary and secondary manifestations of the disease^5^^,^ 7. Corticosteroids slow the progression of muscle weakness and delay some of the complications of the disease, but they do not treat or correct the underlying causes of Duchenne^5^. The use of corticosteroids comes with a high frequency of significant side effects, including behavior change, weight gain, osteoporosis, slowed growth and delayed puberty, and others, which should be monitored by the treating physician.

In an effort to optimize care for DMD patients, international care guidelines for DMD were published in Lancet Neurology in 2009, the generation of which involved more than 80 DMD experts and patient organization representatives^5^^,^ 7. These guidelines highlight the need for a multidisciplinary approach to provide comprehensive care for DMD patients, addressing both the primary and secondary manifestations of the disease and include baseline parameters for key areas, including neurology/steroids, cardiology, pulmonary, physical therapy/physical medicine and rehabilitation, orthopedics, surgical considerations, and psychosocial care. Patient advocacy groups^9^^,^ 10 worked with the TREAT-NMD alliance^11^ to create a family friendly lay version of the guidelines, to help empower parents and patients to advocate for comprehensive care^12^. This family-friendly document, while abbreviated, is still over 30 pages long.

## Competing Interests

**Annemieke Aartsma-Rus**

Employee of LUMC which holds patents on exon skipping, and entitled to royalties as the co-inventor. Ad-Hoc consultant for PTC Global GuidePoint and GLC consultancy which provides remuneration to my institute.

## DMD Imperatives

Comprehensive multidisciplinary care provided by a care team familiar with DMD has been demonstrated to be the best model of care for people living with DMD^7^. However, some individuals live in areas where access to this model is not possible and there is increasing evidence that compliance to the published care standards is patchy at best^13^^,^ 14 ^,^ 15. Furthermore, many DMD patients will visit a team of specialists on a yearly basis and rely on local physicians for their basic care. Since these physicians are generally not DMD specialists, parents and patients need to become DMD experts in order to advocate for their disease specific needs^16^. To assist providers who may have little DMD expertise, in providing patients with comprehensive care, we have generated the “Imperatives for DMD” (Figure 1). This international effort, that involved both clinicians and patient organizations, has resulted in the generation of a one-page document listing the essential elements of comprehensive care for people living with DMD. The mnemonic, DUCHENNE MD, was chosen as an aide-mémoire to the key recommendations in each area of care.

The authors of the Imperatives for DUCHENNE MD, and professionals acknowledged for their participation in this effort, have expertise covering all of the subspecialty areas included in the international care guidelines, and many were involved in the creation of the original guidelines. Each area of the care guidelines was evaluated for consistency with current practices and care. Areas of care that have evolved since the 2009 publication were updated in the Imperatives (i.e., encouraging the use of corticosteroids early (by age 4 years old or as soon as possible after diagnosis if after age 4) and using caution with all anesthesia). Key points from each of the areas of care in the care guidelines were identified and consolidated to develop the single page “Imperatives for DUCHENNE MD.“ While the Imperatives for DUCHENNE MD is not expected to replace the published detailed care guidelines^5^^,^ 7, these simplified care recommendations may serve as an orientation to care for both medical providers not familiar with this population, as well as patients/families.

The imperatives include:

1. Diagnosis

- If developmental delay or elevated liver enzymes, do a creatine kinase (CK) (www.ChildMuscleWeakness.org)
- If male patients have a high CK (CK>800), order full genetic testing for Duchenne muscular dystrophy
- Discuss carrier testing/reproductive options for mother and testing for other family members

A high serum CK is indicative of muscle damage. It is good to bear in mind that skeletal muscle also contains transaminases. The combination of elevated CK and transaminases points to extensive skeletal muscle damage (such as occurs for DMD), rather than liver damage. As discussed above, most children with Duchenne demonstrate delayed motor and/or cognitive development. Therefore, if an elevated CK is found in conjunction with developmental delay and/or elevated transaminases, full genetic testing should be done. If the child is diagnosed with DMD, carrier testing for the mother should be discussed. Mothers who have children with Duchenne have a 66% chance of being diagnosed as a carrier^17^. Definitive testing will allow the couple to make informed decisions regarding future family planning.

2. Use Support

- Direct to trustworthy, reliable online resources
- Organize follow up via a comprehensive neuromuscular center with expertise in caring for people living with Duchenne
- Offer contact with patient organizations

Comprehensive multidisciplinary care provided by a care team familiar with Duchenne has been demonstrated to be the best model of care for people living with Duchenne^5^^,^ 7. Parents faced with the diagnosis of Duchenne are understandably shocked, overwhelmed and grieving. Parents should be encouraged to seek support from other parents either through formally established programs or informally through responsible online resources^18^.

3. Corticosteroids

- Start early!
- Discuss the benefits and possible side effects of corticosteroids by age 3 years, or as young as possible
- Evaluate efficacy and manage side effects of corticosteroids at each neuromuscular visit.
- Discuss the rationale for long term steroid management

In the US, the first symptoms of Duchenne are usually noticed at 2.5 years of age; the average of diagnosis, however, is 4.9 years old^19^ while the median age diagnosis is 4 years old of patients in Europe^20^. Initiation of corticosteroids is recommended at least by ages 4 and 6 years, before motor skills begin to decline^7^^,^ 8. In light of these perceptions and recommendations, it was felt that initiating steroids early would increase their benefit (as soon as diagnosed if after age 4-6 years, and before motor skills begin to decline if diagnosed before ages 4-6 years). Discussing the risks and benefits of corticosteroid use by age 3 years (if diagnosed before age 3 years) would give parents and caregivers time to consider the pros and cons of using of this medication before the motor skills begin to decline.

4 Heart

- Cardiology visit with imaging (echocardiogram or cardiac MRI) at diagnosis or by age 6, then every two years until age 10 (or as needed), then annually (or more often if needed)
- Discuss cardiac medications if fibrosis is seen on cardiac MRI, for any decrease in cardiac function decreases from baseline or for heart failure (SF or shortening fraction <28%, EF or ejection fraction <55%)

Due to deterioration of cardiac muscles, those living with DMD are at a high risk of developing dilated cardiomyopathy and/or cardiac arrhythmia, which often present few clinical symptoms until well advanced^7^. Heightened vigilance of Duchenne providers, including EKG, imaging, and staged interventions when appropriate, is therefore warranted from diagnosis and performed at least annually after age 10^7^. Female carriers of DMD are also at increased risk of developing cardiomyopathy^21^, and should be screened in early adulthood and followed with echocardiogram or cardiac MRI and EKG every five years^22^.

5. Every Visit:

- Monitor weight
- Assess/discuss diet (healthy eating, calcium, vitamin D)
- Evaluate swallowing/need for intervention
- Treat GERD and constipation as necessary

Those with DMD are at risk of both over- and underweight at different stages of the disease, necessitating regular monitoring and dietary adjustments as appropriate^7^^,^ 23. Chronic steroid use can increase risk of younger patients becoming overweight, while older patients, especially those who develop dysphagia, may be at risk for aspiration and losing weight. GERD (gastro-esophogeal reflux) and constipation are the two most commonly reported gastrointestinal conditions reported by children with DMD, and should be treated if they occur^7^. Adequate Vitamin D and calcium intake are also important in maintaining bone health (see below).

6. Never forget Physical and Occupational therapy, physical medicine and rehabilitation

- Specialized evaluations every 4-6 months
- Discuss contracture prevention (splints, stretches), appropriate exercise, assistive mobility devices (strollers, scooters, wheelchairs) and other assistive devices (beds, arm assistance, lifts, etc.)

Regular stretching, and the use of appropriate splints where necessary, helps to prevent the development of contractures and maintain flexibility of joints in those with DMD^5^. Amongst younger children, stretching helps to preserve ambulation; once non-ambulant, stretching is important to help maintain useful upper body function (particularly the use of arms and hands) for as long as possible. Assistive devices such as resting orthoses can help to prevent upper and lower limb contractures, while wheelchairs (initially manual, to prevent overexertion, but later powered) help to maintain independence and societal participation.

7. Nor Bone density

- If taking steroids, check 25-OH vitamin D prior to starting steroids, then annually
- Supplement vitamin D as needed
- Nutrition discussions of adequate calcium and vitamin D intake
- Discuss measurement of bone density and use of bisphosphonates
- Assess spine for scoliosis at each visit

Those on chronic steroid treatment are at risk of fractures. In DMD vertebral fractures are a particular risk, and can be minimized by monitoring and supplementation of vitamin D^5^. Bone density measurement is recommended, while bisphosphonates may also be appropriate. Scoliosis amongst those not treated with steroids is common in DMD and should be monitored carefully. Where necessary, spinal fixation might be required^7^.

8. Evaluate breathing

- Pulmonary function test at least once while ambulatory and every year after loss of ambulation
- Discuss cough assist when cough peak flow is < 270 liters per minute or if cough becomes weaker (use during respiratory illnesses while ambulatory and daily and as needed after loss of ambulation)
- Discuss nighttime Bi-PAP as needed or when forced vital capacity (FVC) < 30%
- Keep immunizations (including pneumonia and annual flu) up to date
- Treat respiratory infections promptly and aggressively
- Do NOT give supplemental oxygen without monitoring CO2

Respiratory decline progressing to failure is a significant complication of DMD. Preventing pulmonary complications with up to date immunizations, pneumonia and annual influenza vaccine is critical^7^. Pulmonary function should be assessed at least once while ambulatory and at least annually after loss of ambulation, with interventions staged appropriately^24^. Following the loss of ambulation, the onset of pulmonary dysfunction is slow and progressive requiring the use of assistive devices. These assist devices generally progress from cough assistance during respiratory illness, to assistance with daily cough, then nocturnal non-invasive ventilation (NIV) and, progressing to continuous NIV (if required). While NIV is generally preferred, in some cases, both patients and pulmonologists have preferred invasive ventilation. Effective airway clearance is essential for the prevention of atelectasis and pneumonia. A peak cough expiratory flow rate of 270 L/min has been designated as the level at which assistive airway clearance (cough assist) is necessary^24^. Sleep disordered breathing and alveolar hypoventilation are both associated with Duchenne and are often subtle and asymptomatic^24^. For this reason, polysomnography (or overnight pulse oxymetry, only if polysomnography is not available) and the use of BiPAP (bi-level positive airway pressure) is recommended if clinically indicated and/or when the FVC < 30%^7^.

9. Mental Health

- Assess adjustment, coping, behavioral and emotional disorder and social isolation for the patient and family at each visit
- Screen for learning disability, speech and language problems, attention deficit disorder (ADD), attention deficit and hyperactivity disorder (ADHD), autism and obsessive compulsive disorder (OCD)
- Neurocognitive evaluation done at diagnosis and prior to formal schooling; screening/management as needed
- Discuss the need for individualized/special educational plan

Comprehensive medical care must also include support for the patient and family’s psychosocial wellbeing^5^. Those with DMD and their families face challenges shared by those living with other chronic, life-limiting conditions, which may include marginalization, social isolation, depression, anxiety, and others^5^. Patients with Duchenne have been found to be at increased risk for coexisting issues, such as learning difficulties, speech and language problems, and ADD/ADHD/OCD, which may compound these challenges. Psychosocial difficulties should be treated with the same interventions as used in the general population to maximize outcomes and enable full social participation. Improvements in medical care have created a new population of adults living with Duchenne. In light of this change, health, education and social services must be encouraged to provide lifelong support to those living with Duchenne^25^.

10. Do:

- Have patients/parents carry a copy of their last visit/note summary (including medications and neuromuscular contact information) and a Duchenne emergency card with them at all times.
- Use caution with all anesthesia; avoid succinylcholine

We recommended that patients and families keep copies of their medical information, and key information about DMD (in the form of an Alert Card or similar), with them at all times. As DMD is a rare condition, non-specialist medical personnel are often unfamiliar with best-practice care or absolute contraindications. Particularly during an emergency, having this information to hand can prevent inappropriate and potentially life-threatening treatment^13^.

There has been general concern in the Duchenne community regarding the use of volatile agents (inhaled agents used for general anesthesia) and the risk of rhabdomyolysis, however the use of these agents and the subsequent development of rhabdomyolysis and hyperkalemia unrelated to malignant hyperthermia remains controversial^26^. In addition, rhabdomyolysis has been reported with the use of non-triggering agents^26^^,^ 27 ^,^ 28. Therefore, the group has recommended that patients be monitored when any anesthesia is used. The use of succinylcholine (a depolarizing agent), however, remains an absolute contraindication in this population^26^^,^ 27.

Notably, most of these Imperatives are in agreement with the aforementioned care guidelines. There are, however, two imperatives that have been updated with regards to new evidence and current expert opinion: the use of corticosteroids and caution with anesthesia.

## Conclusion

“Imperatives for Duchenne MD” is a very focused and brief snapshot of the essential components of comprehensive DMD care as described in the CDC care considerations. This information is meant for health care providers, but can also be used by patients and parent advocating for comprehensive care for themselves or their children. The document has so far been translated into 18 languages by patient organizations and professional volunteers around the world through the TREAT-NMD Alliance.

More detailed information for each area of care and the care required for each stage of Duchenne, as well as the complete publication of the care guidelines are available both online and in print^5^^,^ 7. These publications and the Family Friendly version of the care guidelines are available at the TREAT-NMD website as well.

## Supporting Organizations

**Parent Project Muscular Dystrophy (PPMD)**

Parent Project Muscular Dystrophy (PPMD, www.parentprojectmd.org)^9^ is the largest nonprofit organization in the United States focused entirely on Duchenne. Started in 1994 by Pat Furlong, PPMD takes a comprehensive approach in the fight against Duchenne—funding research, raising awareness, promoting advocacy, connecting the community, and broadening treatment options. PPMD’s care objectives are to identify gaps in care for people with Duchenne and work toward solutions, and to work with clinicians and other health care professionals across the globe to ensure all Duchenne patients have access to optimal care.

**United Parents Project Muscular Dystrophy (UPPMD)**

Duchenne Parent Organizations combine their strength through the worldwide network organization UPPMD (www.uppmd.org)^10^. UPPMD is dedicated to finding a cure and viable treatments for DMD, to promoting good standards of care and to informing parents around the globe. There is a compelling need to ensure that wherever they are in the world, the children suffering from this disease can benefit from a standard of care that is informed by the best practice of the best clinicians from all over the world.

**TREAT-NMD**

The TREAT-NMD Alliance (www.treat-nmd.eu)^11^ is an international translational network dedicated to the neuromuscular field, which brings together researchers, clinicians, patient advocacy organizations. Initially funded as a “Network of Excellence” by the European Union’s 6th Framework Programme (FP6) (2007-2011), it has been maintained and broadened to become a global alliance. TREAT-NMD addresses bottlenecks in the translational process of therapy development for rare, inherited neuromuscular disorders. It provides and links infrastructure tools such as patient registries, biobanks, and standards of care. The global governance structure of TREAT-NMD reserves one third of the Executive Committee positions for patient representatives.

## Figures

**Imperatives for DUCHENNE MD: a guide for providers. A one-page summary of the imperative components of comprehensive multidisciplinary care for patients with Duchenne muscular dystrophy.**
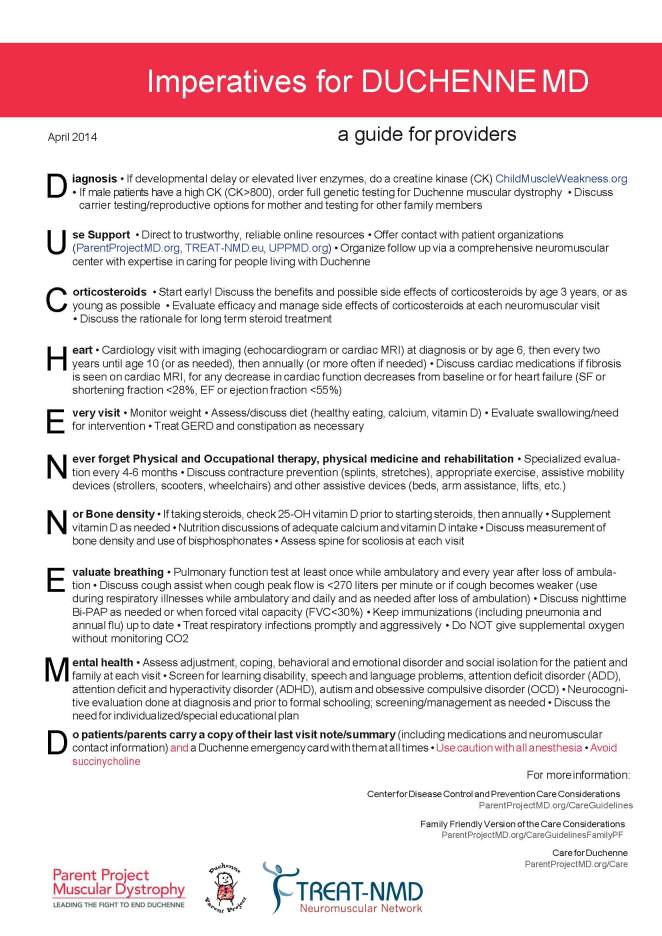

## References

[1] Emery AEH. The muscular dystrophies. 2002;359:687-695 10.1016/S0140-6736(02)07815-7 10.1016/S0140-6736(02)07815-7 11879882

[2] Emery AEH. Population frequencies of inherited neuromuscular diseases—A world survey. 1991;1:19-29 10.1016/0960-8966(91)90039-U 10.1016/0960-8966(91)90039-u 1822774

[3] Mah JK, Korngut L, Dykeman J, Day L, Pringsheim T, Jette N. A systematic review and meta-analysis on the epidemiology of Duchenne and Becker muscular dystrophy. 2014;24:482-491 10.1016/j.nmd.2014.03.008 10.1016/j.nmd.2014.03.008 24780148

[4] Darras BT, Miller DT, Urion DK. Dystrophinopathies. ed. GeneReviews: University of Washington, Seattle, Seattle (WA), 1993.

[5] Bushby K, Finkel R, Birnkrant DJ, et al. Diagnosis and management of Duchenne muscular dystrophy, part 1: diagnosis, and pharmacological and psychosocial management. Lancet Neurology 2010;9:77-93 10.1016/s1474-4422(09)70271-6 10.1016/S1474-4422(09)70271-6 19945913

[6] Cyrulnik SE, Fee RJ, Batchelder A, Kiefel J, Goldstein E, Hinton VJ. Cognitive and adaptive deficits in young children with Duchenne muscular dystrophy (DMD). 2008;14:853-861 10.1017/S135561770808106X 10.1017/S135561770808106X 18764980

[7] Bushby K, Finkel R, Birnkrant DJ, et al. Diagnosis and management of Duchenne muscular dystrophy, part 2: implementation of multidisciplinary care. Lancet Neurology 2010;9:177-89 10.1016/s1474-4422(09)70272-8 10.1016/S1474-4422(09)70272-8 19945914

[8] Eagle M, Bourke J, Bullock R, et al. Managing Duchenne muscular dystrophy – The additive effect of spinal surgery and home nocturnal ventilation in improving survival. 2007;17:470-475 10.1016/j.nmd.2007.03.002 10.1016/j.nmd.2007.03.002 17490881

[9] Parent Project Muscular Dystrophy. Parent Project Muscular Dystrophy Website. Available from: http://www.parentprojectmd.org.

[10] United Parent Projects Muscular Dystrophy. United Parent Projects Muscular Dystrophy Website. [Available from: http://www.uppmd.org]

[11] TREAT-NMD Alliance. TREAT-NMD Alliance Website ; Available from: http://www.treat-nmd.eu.

[12] MDA, PPMD, TREAT-NMD, UPPMD. The Diagnosis and Management of Duchenne Muscular Dystrophy: A Guide for Families. 2010; Available from: http://www.treat-nmd.eu/dmd-care-family.

[13] Cripe L, Kinnett K. Transforming Duchenne Care: meeting 25-26 June 2012, Ft. Lauterdale, Florida, USA. 2013; 8:690-5 10.1016/j.nmd.2013.05.002. 10.1016/j.nmd.2013.05.002 23770103

[14] Budych K, Helms TM, Schultz C. How do patients with rare diseases experience the medical encounter? Exploring role behavior and its impact on patient–physician interaction. 2012;105:154-164 10.1016/j.healthpol.2012.02.018 10.1016/j.healthpol.2012.02.018 22464590

[15] Roger S, Woods K, Bladen C, et al. Adult care of Duchenne muscular dystropy in the UK. J Neuro. 2014; e-publication available at http://link.springer.com/article/10.1007%2Fs00415-014-7585-3 10.1007/s00415-014-7585-3 PMC4363521 25536903

[16] Salzman B, Collins L, Hajjar ER. Chronic Disease Management: The Changing Landscape of Primary Care. 2012;39:xv-xxi 10.1016/j.pop.2012.04.002 10.1016/j.pop.2012.04.002 22608877

[17] Edwards JH. The population genetics of Duchenne: natural and artificial selection in Duchenne muscular dystrophy. 1986;23:521-530 10.1136/jmg.23.6.521 10.1136/jmg.23.6.521 PMC1049833 3806638

[18] Poysky J, Kinnett K. Facilitating family adjustment to a diagnosis of Duchenne muscular dystrophy: April 24–25, 2008, Miami, Florida. 2009;19:733-738 10.1016/j.nmd.2009.07.011 10.1016/j.nmd.2009.07.011 19736011

[19] Romitti P, Puzhankara S, Mathews K, et al. Prevalence of Duchenne/Becker muscular dystrophy among males aged 5-24 years-four states, 2007. 2009;58:1119-1122 19834452

[20] Kole A, Faurisson F, Mavris M. The Voice of 12,000 Patients: Experiences and Expectations of Rare Disease Patients on Diagnosis and Care in Europe. 2009 Available from:: http://www.eurordis.org/IMG/pdf/voice_12000_patients/EURORDISCARE_FULLBOOKr.pdf.

[21] Hoogerwaard EM, van der Wouw PA, Wilde AAM, et al. Cardiac involvement in carriers of Duchenne and Becker muscular dystrophy. 1999;9:347-351 10.1016/S0960-8966(99)00018-8 10.1016/s0960-8966(99)00018-8 10407858

[22] Van Westrum SMS, Hoogerwaard EM, Dekker L, et al. Cardiac abnormalities in a follow-up study on carriers of Duchenne and Becker muscular dystrophy. 2011;77:62-66 10.1212/WNL.0b013e318221ad14 10.1212/WNL.0b013e318221ad14 21700587

[23] Martigne L, et al. Natural evolution of weight status in Duchenne muscular dystrophy: a tetrospective autid. Gr J Nutr.2011;10: 1486-1491. 10.1017/S0007114510005180 21272404

[24] Finder JD, Birnkrant D, Carl J, et al. Respiratory care of the patient with Duchenne muscular dystrophy: ATS consensus statement. American journal of respiratory and critical care medicine 2004;170:456-65 10.1164/rccm.200307-885ST 10.1164/rccm.200307-885ST 15302625

[25] Gibson BE, Zitzelsberger H, McKeever P. ‘Futureless persons’: shifting life expectancies and the vicissitudes of progressive illness. 2009;31:554-568 10.1111/j.1467-9566.2008.01151.x 10.1111/j.1467-9566.2008.01151.x 19220805

[26] Cripe LH, Tobias JD. Cardiac considerations in the operative management of the patient with Duchenne or Becker muscular dystrophy. 2013;23:777-784 10.1111/pan.12229 10.1111/pan.12229 23869433

[27] Gurnaney H, Brown A, Litman RS. Malignant Hyperthermia and Muscular Dystrophies. 2009;109:1043-1048 10.1213/ane.0b013e3181aa5cf6 10.1213/ane.0b013e3181aa5cf6 19762730

[28] Wang J, Stanley T. Duchenne muscular dystrophy and malignant hyperthermia - two case reports. Can Anaesth Soc J 1986;33:492-497 10.1007/BF03010977 10.1007/BF03010977 3742323

